# Subgroups in Late Adulthood Are Associated With Cognition and Wellbeing Later in Life

**DOI:** 10.3389/fpsyg.2021.780575

**Published:** 2021-12-01

**Authors:** Tulsi A. Radhoe, Joost A. Agelink van Rentergem, Almar A. L. Kok, Martijn Huisman, Hilde M. Geurts

**Affiliations:** ^1^Dutch Autism and ADHD Research Center (d’Arc), Department of Psychology, Brain and Cognition, University of Amsterdam, Amsterdam, Netherlands; ^2^Department of Epidemiology and Data Science, Amsterdam Public Health Research Institute, Amsterdam UMC – Location VU University Medical Center, Amsterdam, Netherlands; ^3^Amsterdam UMC, Vrije Universiteit Amsterdam, Department of Psychiatry, Amsterdam Public Health, Amsterdam, Netherlands; ^4^Amsterdam UMC, Vrije Universiteit Amsterdam, Department of Sociology, Amsterdam Public Health, Amsterdam, Netherlands; ^5^Leo Kannerhuis (Youz/Parnassia Groep), Amsterdam, Netherlands

**Keywords:** aging, subgroups, heterogeneity, external validity, predictive validity, longitudinal stability, wellbeing

## Abstract

**Objectives:** In this study, we aim to discover whether there are valid subgroups in aging that are defined by modifiable factors and are determinant of clinically relevant outcomes regarding healthy aging.

**Method:** Data from interviews were collected in the Longitudinal Aging Study Amsterdam at two measurement occasions with a 3-year interval. Input for the analyses were seven well-known vulnerability and protective factors of healthy aging. By means of community detection, we tested whether we could distinguish subgroups in a sample of 1478 participants (T1-sample, aged 61–101 years). We tested both the external validity (T1) and predictive validity (T2) for wellbeing and subjective cognitive decline. Moreover, replicability and long-term stability were determined in 1186 participants (T2-sample, aged 61–101 years).

**Results:** Three similar subgroups were identified at T1 and T2. Subgroup A was characterized by high levels of education with personal vulnerabilities, subgroup B by being physically active with low support and low levels of education, and subgroup C by high levels of support with low levels of education. Subgroup C showed the lowest wellbeing and memory profile, both at T1 and T2. On most measures of wellbeing and memory, subgroups A and B did not differ from each other. At T2, the same number of subgroups was identified and subgroup profiles at T1 and T2 were practically identical. Per T1 subgroup 47–62% retained their membership at T2.

**Discussion:** We identified valid subgroups that replicate over time and differ on external variables at current and later measurement occasions. Individual change in subgroup membership over time shows that transitions to subgroups with better outcomes are possible.

## Introduction

Positive aging trajectories translate to higher wellbeing and better physical functioning, and lower health care costs for society (Bowling and Dieppe, 2005; Kok et al., 2017) but it is difficult to predict who will have such a positive trajectory, and who will require more support. Large differences exist in how people age (Hayden et al., 2011; Lowsky et al., 2013). The aging patterns observed in group studies are also diverse. Some groups show strong average decline in functioning as people age, others show less decline or even no decline at all (Grundy and Bowling, 1999; Wilson et al., 2002; Kok et al., 2017). As there are large individual differences it might be more informative to focus on aging patterns of subgroups instead of across the general population. If we overlook these interindividual differences, we may falsely conclude that there is no change due to aging, while in fact different outcomes (e.g., stability, decline, or increase over time) apply to different subgroups. Thus, adopting a subgrouping approach provides us with information that otherwise would have been overlooked. In this study, we aim to identify subgroups of aging adults that are either more or less likely to experience subjective cognitive decline and decreased wellbeing; currently, and in the future. We use easy to measure and modifiable proxies of well-known vulnerability and protective factors for healthy mental aging as input variables.

A lack of subjective cognitive decline and a high wellbeing are both typical characteristics of healthy mental aging. People associate aging with a decrease in memory, and subjective cognitive decline is predictive of cognitive impairment and dementia in older adults (Geerlings et al., 1999; Jonker et al., 2000). However, aging does not necessarily lead to cognitive decline (Jonker et al., 2000; Lima-Silva and Yassuda, 2009; Silva et al., 2014). Similarly, while wellbeing tends to decrease with age, the speed and associated risk factors vary across studies and groups (Jivraj et al., 2014; Lukaschek et al., 2017). Whether wellbeing decreases over age also depends on which aspect of wellbeing is addressed; for example, life satisfaction is generally high in old age (e.g., Charles and Carstensen, 2010). Maintaining wellbeing is often a primary goal for healthcare of older adults as they age. In this study we use wellbeing as an umbrella term reflecting mental, social, physical and spiritual wellbeing, and personal circumstances, activities and functioning (Linton et al., 2016).

Subjective cognitive decline and wellbeing in older age could be affected by behavior, psychological, and/or social factors (e.g., physical activity, alcohol use, social support) as well as (neuro)biology (e.g., genetics, brain structure) (see for example Chen et al., 2014; Eva et al., 2015). In this study, we focus on factors for which easy to administer and inexpensive measures are available. We particularly focus on those factors that could be influenced by psychological interventions and/or environmental changes. By focusing on variables that are easy to measure and modifiable in nature rather than factors that are expensive to measure and fixed, we hope to discover subgroups that can inform and guide clinical practice and preventive health services.

The relationship between risk and protective factors and aging outcomes is complex. Many different factors have been shown to influence aging outcomes, some of which may be intercorrelated and may be reflective of a more general common risk factor, while other factors may independently affect outcomes (Christensen et al., 2001). Furthermore, some factors may directly influence outcomes, while other factors affect outcomes in their interaction (e.g., Windsor et al., 2020; Sauter et al., 2021). The advantage of examining factors together is that we can reduce this complexity. By taking a multivariate approach, we reduce the complexity of interacting individual differences on a large number of variables to a limited number of interpretable profiles.

In the literature, different types of input variables have been used to construct subgroups, which has resulted in varying numbers of subgroups with varying characteristics. For example, neurocognitive test variables were used to identify three latent classes of cognitive performance in older individuals (Costa et al., 2013). Another study identified five subgroups of older adults using social engagement activity patterns (Croezen et al., 2009). Nine profiles of functional status were identified using measures of psychological functioning in older adults as part of the Berlin Aging Study (Smith and Baltes, 1997). When subgroups are defined by non-modifiable variables, their usefulness is inherently limited, as the subsequent subgroups are also more or less set in stone. Moreover, the validity of most obtained subgroups and their stability over time remains an open question. A lack of systematic validation of subtypes will lead to a proliferation of subtypes of questionable utility (Agelink van Rentergem et al., 2021).

In this study, we perform three subgroup validation techniques. First, we assess external validity by investigating whether subgroups differ in subjective cognitive decline and wellbeing. Second, we assess predictive validity by determining whether subgroups differ in subjective cognitive decline and wellbeing after 3 years. Third, we assess longitudinal stability of subgroups by repeating the community detection analysis on data collected after 3 years. Specifically, we assess whether the same number of subgroups is identified after 3 years, and whether subgroup profiles are the same. With these validation techniques, we assess whether subgroups generalize to other domains, have predictive value for other domains, and are stable over time.

## Materials and Methods

### Study Sample

Data was requested from the Longitudinal Aging Study Amsterdam (LASA), an ongoing prospective study of older adults living in Netherlands (Hoogendijk et al., 2016). LASA’s objective is to investigate the determinants, trajectories, and consequences of physical, emotional, cognitive, and social functioning related to aging (Huisman et al., 2011). The study is based on a nationally representative sample of adults aged 55–85 years (born in 1908–1937), recruited from municipal registries in Netherlands, who completed interviews at home. In 1992, the first 3107 adults participated (cooperation rate 62%). Since baseline, measurements were repeated about every 3 years. In 2002–2003, a refresher sample of 1002 participants aged 55–65 (born in 1938–1947) was added. Participants from the first and refresher sample were measured together from the regular follow-up measurement of 2005–2006 onward. LASA data are available for research and can be requested by submitting an analysis proposal to the LASA Steering Group (see www.lasa-vu.nl for more info).

For our study, data from the seventh and eight wave of data collection were included. See Supplementary Section 1 for the names of the specific data files used in the current study. 1601 participants were included from the seventh wave (2008–2009, T1), of whom 1478 were analyzed after removing observations with too many missing values (see below). 1275 participants were included from the eighth wave (2011–2012, T2), of whom 1186 were analyzed. The T1-sample (675 men, 803 women) had a mean age of 73 years (*SD* = 8.29, range = 61–101). The T2-sample (537 men, 649 women) had a mean age of 75 years (*SD* = 7.58, range = 64–100).

### Measures

Selection of cluster variables was guided by their (1) relation to cognitive decline and/or wellbeing (MacDonald et al., 2011; Goh et al., 2012; Beydoun et al., 2014; Chen et al., 2014; Prenderville et al., 2015), (2) individual differences in the aging population, (3) quick and large-scale measurement through self-report, and (4) modifiability. The variables fit with these guiding principles to varying degrees. For example, the impact of negative life events may be indirectly modifiable as their effect can be modified through interventions; one’s education is unlikely to change at older age but may be modifiable earlier in life; alcohol use is directly modifiable. Both education and excessive alcohol use have recently been named among the most important modifiable factors with respect to increased risk of dementia (Livingston et al., 2020). In total, we included seven cluster variables.

#### Cluster Variables

A detailed description including psychometric properties can be found in the Supplementary Section 2.

##### Negative Life Events

Negative life events were evaluated with questions from the life event inventory (Tennant and Andrews, 1976). Participants reported whether they had experienced 12 different negative life events in the past 3 years (see Supplementary Section 2). We calculated a sum score that ranged from 0 (no negative life events) to 12 (many negative life events) (see also Comijs et al., 2011). Negative life events have strong associations with depressive symptoms and lower wellbeing (Kraaij et al., 2002). Resources such as social network, education and health status are inversely associated with the impact of negative life events later in life (Jopp and Schmitt, 2010).

##### Education

Responses on educational level were translated into years of education and ranged from 5 (elementary school not completed) to 18 (university education). Lower educational attainment is associated with subjective cognitive decline and is a strong predictor of dementia (Beydoun et al., 2014; Chen et al., 2014).

##### Alcohol Use

Participants reported the number of days per week on which they drink alcohol and the number of alcoholic consumptions they drink each time. The possible number of alcoholic consumptions per week ranged from 0 (no alcoholic drinks) to 77 (or more) (see for a similar approach Pluijm et al., 2006; Comijs et al., 2012). Alcohol use is related to cognitive decline (Mintzer, 2007; Heffernan, 2008) and can be targeted in interventions (Platt et al., 2016).

##### Physical Activity

Physical activity was assessed during an interview using the LASA Physical Activity Questionnaire (Stel et al., 2004). Participants reported how often and for how long they performed various physical activities during the 2 weeks prior to the interview (see Supplementary Section 2). We calculated the total time in minutes. A higher level of physical activity is associated with less cognitive decline and predicts wellbeing in older adults (McAuley et al., 2000; Beydoun et al., 2014; Kadariya et al., 2019). Also, physical activity levels can be increased through interventions (Müller-Riemenschneider et al., 2008; Greaves et al., 2011).

##### Emotional and Instrumental Support Received

We asked participants about people they are regularly in touch with and are important to them (Van Tilburg, 1998). Participants reported the supportive emotional and instrumental exchanges with the nine most important network members, excluding the partner (see Supplementary Section 2). Questions were answered with four response options, ranging from “never” to “often.” Sum scores for emotional support received and instrumental support received were calculated varying between 0 (low level of support) and 36 (high level of support). Leading a socially active life and receiving sufficient social support are related to a higher wellbeing later in life (Yaffe et al., 2009; Gerstorf et al., 2016). Interventions for social support can be effective in increasing one’s perceived level of social support (Hogan et al., 2002).

##### Sense of Mastery

Mastery refers to the extent to which people view themselves as being in control of the forces that affect their lives in important ways (Pearlin et al., 1981). Mastery was assessed by the Pearlin Mastery Scale, consisting of seven items rated on a five-point scale ranging from “strongly disagree” to “strongly agree” (Pearlin and Schooler, 1978). We calculated a sum score varying between 7 and 35. Higher ratings indicate a stronger internal locus of control. A high level of mastery, or stronger internal locus of control, is related to a better memory performance and higher wellbeing (Amrhein et al., 1999; Verhaeghen et al., 2000; Robinson and Lachman, 2017). Mastery and self-efficacy can be increased through interventions (Mathisen and Bronnick, 2009).

#### External Validators

##### Subjective Wellbeing

Subjective wellbeing was measured with three different self-report questionnaires. First, we assessed satisfaction with life by two questions defined by Van Zonneveld (1961). The first question asks about satisfaction with current life, the second about satisfaction with life as a whole. Both questions have five response categories ranging from “very dissatisfied” to “very satisfied.” We calculated a sum score ranging from 2 (low satisfaction with life) to 10 (high satisfaction).

Second, health-related quality of life was measured by the EuroQoL five dimensional questionnaire (EQ-5D). It consists of five questions and a visual analog scale. Responses on these items were converted into a weighted health state index according to the Time Trade OFF method (Dolan, 1997) ranging from 0 (low) to 1 (high).

Third, we measured functional health and wellbeing by the Short Form 12 (SF-12) health survey, a subset of the larger SF-36 (Ware et al., 1996). Sum scores were calculated for two summary scales of the SF-12, the Physical Component Summary (PCS) and the Mental Component Summary (MCS).

##### Subjective Cognitive Decline

We asked whether participants experience memory complaints during a broader medical interview. This question is reliable in identifying people vulnerable to cognitive impairment or dementia (Geerlings et al., 1999). A score of 1 indicated memory complaints and a score of 0 indicated no complaints.

#### Additional Descriptive Variables

The following additional variables were not used as cluster variables or external validators but were used to further describe the subgroups: Age, country of origin, marital status and sex (all measured at the first wave of LASA), medication use, household composition, fluid intelligence, depression diagnosis, anxiety diagnosis, and ADHD symptoms (all measured at the same occasion as the cluster and external validation variables). These last measures were included to characterize internalizing and externalizing problems. See Supplementary Section 3 for a more detailed description of the measurement instruments and descriptive analyses.

### Statistical Analyses

The analysis plan was preregistered at AsPredicted.org (AsPredicted number: 27409, https://aspredicted.org/7np2t.pdf).

#### Missing Data

If participants had less than two missing values on the seven cluster variables, we included them in the analysis. We considered 10% an acceptable amount of missing data for imputation (Bennett, 2001). For mastery, we recoded a maximum of one missing value to the median of the participant’s responses on the other mastery items. For negative life events, we recoded a maximum of one missing value to 0 (i.e., the event did not occur in the past 3 years).

If participants had missing values on two or more of the seven cluster variables, they were excluded from analysis. At T1, 123 participants out of 1601 were excluded due to missing data, which led to 1478 analyzed participants. At T2, 89 participants out of 1275 were excluded, which led to 1186 analyzed participants.

#### Community Detection Analysis

To establish subgroups, we used a state-of-the art method, called community detection. Community detection is a non-parametric clustering method that stems from the mathematical discipline of graph theory (Dorogovtsev and Mendes, 2003; Newman, 2010). With this method, we take into account the multivariate structure of different risk and protective factors of subjective cognitive decline and wellbeing. People with similar profiles on the input variables have a higher likelihood of being assigned to the same subgroup than people with dissimilar scores. Research so far suggests that the added value of this novel method compared to Latent Profile Analysis —which is commonly used to investigate heterogeneity— could be the identification of subgroups that are more stable over time with improved clinical predictive value (Blanken et al., 2020).

Our goal was to identify the community structure in a network of people. Cluster variables were first standardized to *z-*scores, to prevent differences in measurement scales from affecting results. We then created a pairwise Pearson correlation matrix containing relationships between scoring patterns of all pairs of individuals (see for similar approach Karalunas et al., 2014). Pairs of individuals whose scoring patterns on cluster variables are similar show a high correlation in this matrix.

A network was created containing nodes, which represent people in this case, connected by edges, which are correlations between people in this study. A community is a subgraph in the larger network, where the number of internal edges (within the community) is larger than the number of external edges (between communities) (Fortunato and Hric, 2016). In other words, nodes in a community have a higher likelihood of connecting to each other than to nodes from other communities (Barabási and Pósfai, 2016).

Multiple algorithms can be applied to identify communities. We had three criteria for the algorithm. First, it should deal with weighted edges, i.e., correlations. Second, it should deal with positive and negative correlations. If we would only include positive correlations, we may include people who are dissimilar in the same community, which interferes with our goal of creating homogeneous subgroups. Third, it should not result in overlapping communities. If people belong to multiple communities, we cannot transfer them from one community to another (more favorable) community. The Spinglass algorithm meets these criteria and was selected (Reichardt and Bornholdt, 2006). This algorithm rewards internal edges between nodes of the same subgroup. Second, it penalizes missing edges between nodes in the same subgroup. Third, it penalizes existing edges between different subgroups. Fourth, it rewards non-existing edges between different subgroups. We assigned equal importance to existing edges and non-existing edges, and to positive and negative weights, between individuals, by setting the γ-parameter to 1. We also calculated the modularity index *Q*, which measures the quality of the assignment of nodes into communities (Newman and Girvan, 2004). The maximum value is *Q* = 1, indicating a strong community structure. In practice, most values range from 0.3 to 0.7.

#### Descriptive Analyses of Subgroups

We performed six ANOVAs and six Pearson’s χ^2^ tests on additional variables to describe the identified subgroups. These analyses are described in more detail in Supplementary Section 3.

#### Subgroup Validation

To assess external validity of subgroups, we compared subgroups on wellbeing and subjective cognitive decline measured at T1. To assess predictive validity, we compared subgroups on these same variables, this time measured at T2. We considered the subgrouping solution meaningful if subgroups differed significantly on these external variables, using ANOVA and logistic regression, with subgroup membership at T1 as the independent variable.

To assess the longitudinal stability of the subgroups identified at T1, we repeated the community detection analysis on data collected at T2. We created a contingency table of subgroup assignment at T1 versus assignment at T2. We performed a χ^2^ test for association between subgroup assignment at these time points.

## Results

### Community Detection Analysis

**FIGURE 1:**
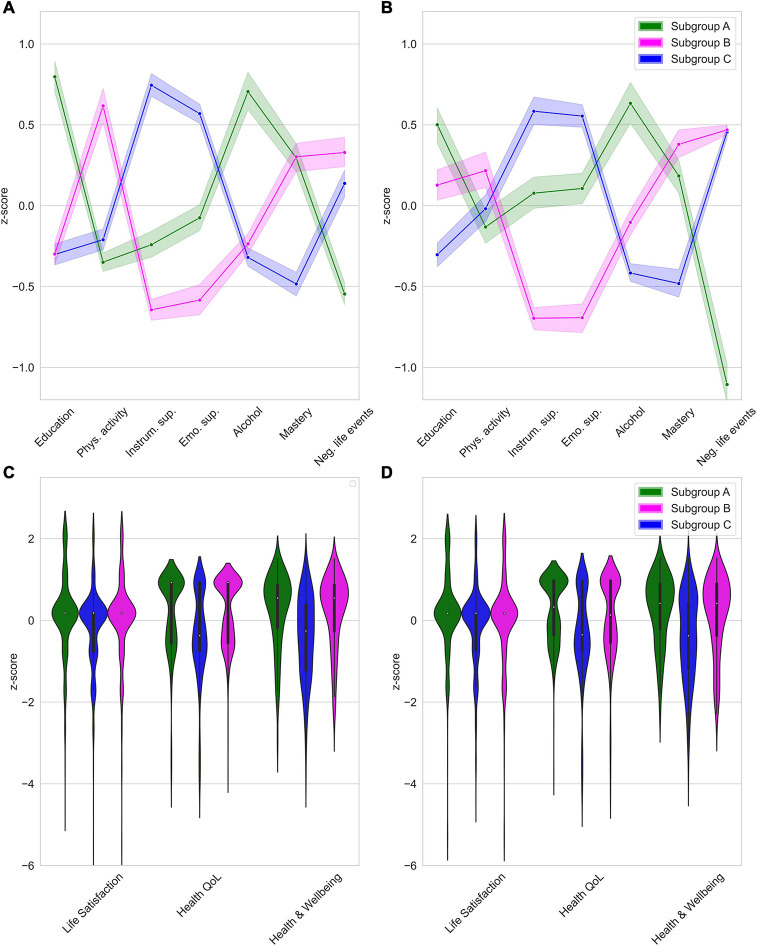
**(A)** Subgroup profiles at T1, **(B)** Subgroup profiles at T2, **(C)** External validation (T1), **(D)** Predictive validation (T2). Scores as shown as *z*-scores based on the total sample mean. Shaded area represents 95%-confidence interval.

Three distinct subgroups were identified. The modularity index indicated weakly defined communities, *Q* = 0.26. Figure 1A depicts subgroup profiles on the cluster variables at T1. Table 1 presents test statistics for the findings described below.

**TABLE 1:** Raw cluster variable scores and descriptives for each of the three community detection-based subgroups formed on T1 data (*N* = 1478).

	**Subgroup**						
	**Subgroup A**	**Subgroup B**	**Subgroup C**				

**Variable**	***N* = 435**	***N* = 486**	***N* = 557**	**Test statistic (*df*)**	**Subgroup C vs. Subgroup B (*Z)***	**Subgroup A vs. Subgroup B (*Z)***	**Subgroup A vs. Subgroup C (*Z)***
**Descriptive variables**							
Age *M*(*SD*)	70.75 (7.58)	72.30 (8.32)	75.02 (8.30)	*F*(2,1475) = 35.66^***^	5.54^***^	−2.81^***^	−8.27^***^
# household members *M*(*SD*)	0.80 (0.56)	0.77 (0.59)	0.66 (0.63)	*F*(2,1446) = 7.08^***^	−3.28^***^	0.82	4.03^***^
# medicines *M*(*SD*)	2.63 (2.73)	2.84 (2.66)	4.16 (3.04)	*F*(2,1414) = 42.36^***^	7.16^***^	–1.51	−8.52^***^
Raven *A*-score *M*(*SD*)	10.58 (2.22)	10.18 (1.84)	9.78 (2.55)	*F*(2,1414) = 14.94^***^	–1.78	5.41^***^	7.31^***^
Raven *B*-score *M*(*SD*)	9.04 (2.90)	8.29 (2.82)	7.67 (3.11)	*F*(2,1414) = 25.41^***^	−3.12^**^	4.78^***^	7.96^***^
ADHD-score *M*(*SD*)	0.51 (1.16)	0.45 (0.96)	0.79 (1.38)	*F*(2,1388) = 11.48^***^	4.40^***^	0.40	−3.87^***^
Gender				χ^2^(2) = 84.18^***^			
*N*_male_ (%)	276 (63)	205 (42)	194 (35)				
*N*_female_ (%)	159 (37)	281 (58)	363 (65)				
Country of Origin				χ^2^(2) = 2.17			
*N*_Netherlands_ (%)	431 (99)	485 (99)	554 (99)				
*N*_Other_ (%)	4 (1)	1 (1)	3 (1)				
Current depression^*a*^				χ^2^(2) = 0.52			
*N*_Yes_ (%)	3 (6)	3 (5)	10 (7)				
*N*_No_ (%)	49 (94)	61 (95)	128 (93)				
Lifetime depression^*a*^				χ^2^(2) = 3.10			
*N*_Yes_ (%)	14 (27)	9 (14)	31 (22)				
*N*_No_ (%)	38 (73)	55 (86)	107 (78)				
Lifetime anxiety^*a*^				χ^2^(2) = 3.46			
*N*_Yes_ (%)	10 (20)	21 (36)	37 (28)				
*N*_No_ (%)	41 (60)	38 (64)	95 (72)				
Medication use				χ^2^(2) = 47.24^***^			
*N*_Yes_ (%)	308 (74)	354 (76)	478 (90)				
*N*_No_ (%)	111 (26)	111 (24)	55 (10)				
Marital status				χ^2^(6) = 52.41^***^			
*N*_never married_ (%)	33 (8)	23 (5)	17 (3)				
*N*_married_ (%)	296 (68)	325 (67)	308 (55)				
*N*_divorced_ (%)	35 (8)	38 (8)	45 (8)				
*N*_widowhood_ (%)	71 (16)	100 (20)	178 (34)				
**Cluster variables**							
Phys. activity *M*(*SD*)	117.22 (71.60)	212.09 (120.02)	129.31 (78.69)	*F*(2,1465) = 156.70^***^	−12.29^***^	−13.96^***^	−2.52^**^
Instr. support *M*(*SD*)	13.95 (5.04)	11.39 (4.61)	20.04 (5.25)	*F*(2,1475) = 420.40^***^	22.71^***^	6.41^***^	−15.43^***^
Emo. support *M*(*SD*)	21.85 (6.71)	18.02 (7.80)	26.70 (5.19)	*F*(2,1473) = 227.20^***^	18.25^***^	6.81^***^	−10.67^***^
Alcohol use *M*(*SD*)	14.23 (12.94)	5.93 (7.45)	5.05 (6.98)	*F*(2,1410) = 177.30^***^	−1.97^*^	13.28^***^	15.64^***^
Mastery *M*(*SD*)	25.13 (3.70)	25.25 (4.00)	22.03 (3.70)	*F*(2,1473) = 123.90^***^	−12.84^***^	–0.22	12.23^***^
Neg. events *M*(*SD*)	0.48 (0.63)	1.26 (0.93)	1.09 (0.90)	*F*(2,1461) = 109.30^***^	−2.96^**^	−13.69^***^	−11.25^***^
Education *M*(*SD*)	11.94 (3.65)	9.00 (2.77)	8.99 (2.80)	*F*(2,1475) = 246.30^***^	0.12	15.91^***^	16.30^***^

* *p* < 0.05, ** *p* < 0.01, *** *p* < 0.001.

*^a^Sample sizes are lower for these variables because data are only available for participants who completed a CIDI-interview.*

We refer to the first subgroup (*N*_1_ = 435; 29%) as “*Subgroup A*.” This subgroup had the highest educational level attained, lowest level of physical activity and the highest use of alcohol. These participants experienced fewer negative life events than other subgroups. We labeled the second subgroup (*N*_2_ = 486; 33%) “*Subgroup B*.” This subgroup reported the highest physical activity level and received the lowest levels of emotional and instrumental support. Furthermore, this subgroup experienced the highest number of negative life events compared to the other subgroups. We labeled the third subgroup (*N*_3_ = 557; 38%) “*Subgroup C*.” This subgroup received the highest levels of instrumental and emotional support. This subgroup also reported the lowest sense of mastery.

### Description of Subgroups

Subgroups differed from each other on several descriptive measures at T1 (see Table 1). Participants in Subgroup A were younger and showed higher scores on fluid intelligence which aligns with this being the highly educated subgroup. Moreover, this subgroup contained more men than women, while the other two subgroups contained more women. Participants in the Subgroup C were older and had lower fluid intelligence scores than the other subgroups. Also, they had the highest ADHD-scores, the smallest household composition and used the highest number of medicines. Moreover, this subgroup included a higher number of participants in widowhood and lower number of married participants compared to the other subgroups, which corresponds with the older ages of participants in this subgroup.

### External Validation: Strongly Supported Subgroup C Scored Lower Than Other Subgroups

For external validation, we tested subgroup differences in four preregistered external measures. Results for measures related to wellbeing are presented in Table 2. Figures 1C,D depicts violin plots of the distribution on variables measuring wellbeing (this is not possible for subjective cognitive decline due to binary response categories).

**TABLE 2:** Scores for external (T1) and predictive (T2) validation measures for each of the three community detection-based subgroups formed on T1 data.

	**Subgroup**						
	**Subgr. A**	**Subgr. B**	**Subgr. C**				

**Variable**	***M*(*SD*)**	***M*(*SD*)**	***M*(*SD*)**	***F*(*df*)**	**Subgr. C vs.** **Subgr. B (*Z)***	**Subgr. A vs.** **Subgr. B (*Z)***	**Subgr. A. vs.** **Subgr. C (*Z)***
**External validation**							
Life satisfaction (T1)	0.16 (1.01)	0.06 (1.01)	−0.18 (0.97)	*F*(2,1376) = 14.16^***^	−4.42^***^	1.11	5.48^***^
Health-related QoL (T1)	0.21 (0.94)	0.22 (0.80)	−0.34 (1.10)	*F*(2,1362) = 54.63^***^	−8.46^***^	0.52	8.88^***^
Functional health and wellbeing (T1)	0.25 (0.92)	0.24 (0.88)	−0.41 (1.03)	*F*(2,1211) = 65.53^***^	−9.52^***^	0.34	9.71^***^
**Predictive validation**							
Life satisfaction (T2)	0.14 (0.97)	< −0.01 (1.03)	−0.11 (1.00)	*F*(2,1117) = 5.97^**^	−2.38^*^	2.15^*^	4.49^***^
Health-related QoL (T2)	0.26 (0.79)	−0.06 (1.00)	−0.30 (1.09)	*F*(2,1069) = 30.71^***^	−5.01^***^	2.42^*^	7.35^***^
Functional health and wellbeing (T2)	0.26 (0.85)	0.17 (0.95)	−0.40 (1.06)	*F*(2,975) = 46.54^***^	−7.28^***^	1.00	8.17^***^

* *p* < 0.05, ** *p* < 0.01, *** *p* < 0.001.

On all domains related to wellbeing (i.e., life satisfaction, health-related QoL, and functional health), Subgroup C scored significantly lower than Subgroups A and B. There were no differences in wellbeing domains between Subgroups A and B. Table 3 presents test statistics related to the findings of subjective cognitive decline. Being a member of Subgroup C compared to Subgroup B, multiplied the odds of experiencing subjective cognitive decline by 1.57. Being a member of Subgroup C compared to Subgroup A, multiplied the odds of subjective cognitive decline by 1.46.

**TABLE 3:** Scores related to subjective cognitive decline for external (T1) and predictive (T2) validation for each of the three community detection-based subgroups formed on T1 data.

				**95% CI for Odds Ratio**		
**Subgroup comparison**	***B* (*SE*)**	***df***	***p***	**Lower**	**Odds ratio**	**Upper**
**External validation** **(T1)**						
Subgr. B vs. Subgr. A	−0.07(0.15)	1	0.614	0.70	0.93	1.24
Subgr. C vs. Subgr. B	0.45 (0.13)	1	0.001	1.21	1.57	2.04
Subgr. C vs. Subgr. A	0.38 (0.14)	1	0.005	1.12	1.46	1.91
**Predictive validation (T2)**						
Subgr. B vs. Subgr. A	−0.07(0.15)	1	0.650	0.69	0.93	1.26
Subgr. C vs. Subgr. B	0.33 (0.14)	1	0.021	1.05	1.40	1.85
Subgr. C vs. Subgr. A	0.26 (0.15)	1	0.075	0.97	1.30	1.74

### Longitudinal Stability: A Small Majority Remains in the Same Subgroup

Community detection analysis was repeated using T2 data (*N* = 1186). There was more drop-out from T1 to T2 in Subgroup C compared to the other two subgroups. Compared to participants that did not drop out, the drop-out group was older, used a higher number of medicines and had lower fluid intelligence scores. Attrition from T1 to T2 is further described in Supplementary Section 4.

**FIGURE 2:**
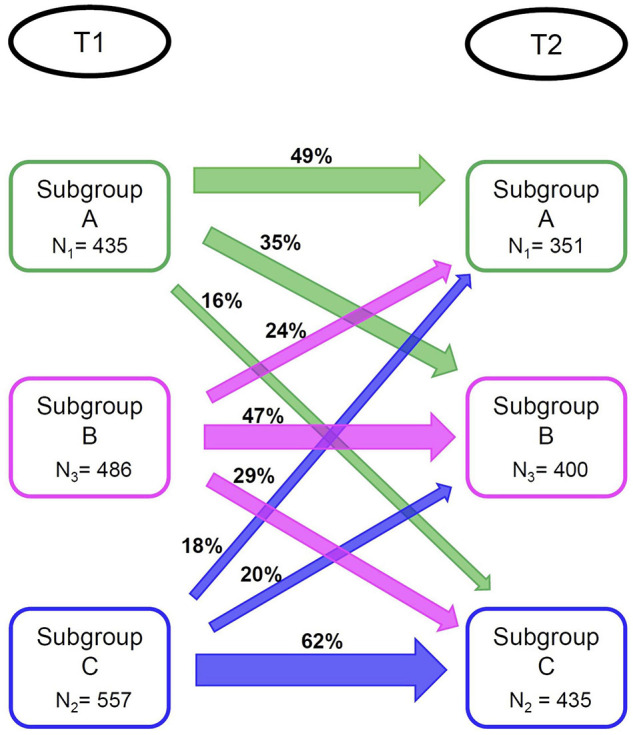
Stability of Subgroup Membership from T1 to T2.

Again, we identified three subgroups (*N*_1_ = 351, *N*_2_ = 435, *N*_3_ = 400). Profiles of the subgroups identified at T2 are presented in Figure 1B. We used the same labeling as for the subgroups formed at T1, since subgroup profiles observed at T2 were highly similar to those observed at T1 (although subgroup differences regarding physical activity were smaller at T2 than at T1). There was a significant association between subgroup membership at T1 and at T2 [*χ*^2^(4) = 229.52, *p* < 0.05]. For specific percentages regarding subgroup membership stability, see Figure 2^1^. While not preregistered, we explored changes in subgrouping variables for those participants whose subgroup membership was not stable over time (see Supplementary Section 6 for supporting figures). Switches to and from Subgroup A seem to be driven by changes in negative life events. Switches between the Subgroup B and Subgroup C were associated with changes in instrumental and emotional support.

### Predictive Validity: All Three Subgroups Differ on External Validators at T2

We used subgroup membership at T1 to predict wellbeing and experience of subjective cognitive decline at T2 to assess the predictive validity (i.e., are the earlier discovered differences on the external validators stable over time). Results are presented in Table 2. Figure 1D depicts violin plots of scores per subgroup on wellbeing variables at T2. On all wellbeing domains (i.e., life satisfaction, health-related QoL, and functional health), Subgroup C scored significantly lower than Subgroups A and B. Subgroup A scored higher than Subgroup B on life satisfaction and health-related QoL, but these subgroups did not differ on functional health at T2. Table 3 presents test statistics related to prediction of experiencing subjective cognitive decline. Being a member of Subgroup C compared to Subgroup B multiplied the odds of experiencing subjective cognitive decline at T2 by 1.40.

## Discussion

In this study, we identified three subgroups of older adults by analysis of protective and vulnerability factors of aging: Subgroup A, characterized by average levels of social support, high alcohol use, low number of experienced negative life events, low physical activity level and high educational level; Subgroup B, characterized by a high physical activity level, low levels of social support, and high number of experienced negative life events; Subgroup C, characterized by high levels of social support and low sense of mastery. We further assessed the validity of these subgroups and their longitudinal stability.

Subgroup C differed from the other subgroups by displaying lower scores on wellbeing and higher odds of experiencing subjective cognitive decline. The other two subgroups were highly similar when focusing on external validators. At the second measurement, Subgroup C was again associated with the most vulnerable profile on the external variables. At this occasion, Subgroup A scored higher on two wellbeing measures than Subgroup B. Repeating the subgrouping analysis at the second measurement occasion yielded the same number and character of subgroups, but just 47–62% of participants retained their subgroup membership over time.

Subgroup C, characterized by high levels of social support, was associated with the lowest cognitive and wellbeing profile at T1 and T2, while social support is often seen as a protective factor for psychological distress (Bøen et al., 2012). The observed low wellbeing and cognitive profile in our study could be explained by necessity of this social support alongside other vulnerabilities adults in this subgroup may experience, such as a low sense of mastery. Since this subgroup also experienced more negative life events, more ADHD symptomatology, and used more medicines, social support may best be seen as a necessity in the face of other vulnerabilities.

Subgroup B, also scoring less favorably on cluster variables (i.e., high number of negative life events), was not associated with a lower wellbeing and memory profile. One explanation for this difference compared to Subgroup C, could be the high sense of mastery experienced by individuals in Subgroup B. If participants in this subgroup feel they are more in control of their lives, they may be more capable to deal with other vulnerabilities, while requiring less social support. This may be associated with a better wellbeing and cognitive profile.

Subgroups seem stable over time, since we identified the same number of subgroups at T2, and subgroup profiles at T1 and T2 were practically identical; although Subgroup B was somewhat less physically active at T2. However, only 47–62% per subgroup retained their membership at T2. Instability in subgroup memberships over time alongside stable subgroup profiles has been indicated in previous studies using community detection (Karalunas et al., 2014; Blanken et al., 2020). Transitions between subgroups over time are also more likely when modifiable cluster factors are included, as in our study. Future research may address whether these factors can be targeted in (clinical) interventions to ultimately transfer people to a subgroup with a more beneficial outcome.

Changes in group membership were primarily driven by changes in negative life events. This may be due to the importance of negative life events, but this variable was also more changeable than other variables because of how it was measured. Participants were asked about negative life events they experienced in the past 3 years, with 3 years between measurement occasions, which means that the same negative life events are not counted twice. To illustrate, if a participant experienced the death of a father in the 3 years prior to T1, this negative life event cannot be experienced again in the 3 years prior to T2, while for example education is the same between T1 and T2. Therefore, changes in the number of negative life events are more likely than changes in education, and thus are also more likely to drive changes in subgroup membership. Negative life events are perhaps most interesting as they were influential in subgroup membership changes, but may only be indirectly modifiable.

The implication of our results is that aging research should consider investigating these subgroups separately. Broad statements on the relationship between a particular risk factor, or on a trend of worsening cognitive functioning, may only be true for a third, or two-thirds, of the elderly population. Claiming that a particular result holds for the entire population based on an analysis that does not take into account the individual differences that we explored here may be unnecessarily concerning to those who are unlikely to encounter these problems. Conversely, researchers may be unable to detect effects of certain factors at the level of the population, while the impact may be large within one of the subgroups. In adjusting care, these effects are particularly important, so we do not want these to be overlooked.

Irrespective of the strengths of this study, there are some limitations. Firstly, we asked participants whether they experienced memory complaints (“yes” or “no”). However, one will have a more comprehensive view of cognitive decline when using a more sensitive measure for subjective cognitive decline like the Cognitive Failures Questionnaire (Broadbent et al., 1982), and/or include a neuropsychological test for objective memory problems. To investigate whether using a neuropsychological test would have changed results, we additionally included data from a verbal learning test (Klaming et al., 2017) to investigate whether subgroups differed in their objective episodic memory as well (not preregistered). They did differ in total recall, both in external validation at the same measurement occasion [*F*(2,1365) = 16.843, *p* < 0.001], and in predictive validation at the second measurement occasion [*F*(2,1123) = 12.632, *p* < 0.001]. Subgroup C again had the worst memory scores. Therefore, the results do not seem to be limited to self-report.

Secondly, while the current attrition rates are congruent with those of other longitudinal studies (Fischer et al., 2001; Young et al., 2006), we cannot exclude the possibility that the drop-out group would form an additional subgroup at T2 when included. Compared to the group that participated at both time points, the drop-out group was older, used more medicines, and had lower fluid intelligence scores. The drop-out group included more participants from the most vulnerable subgroup, associated with the lowest wellbeing and cognitive profile. Therefore, attrition may have been somewhat differential. Thirdly, some might argue that we should have corrected for baseline performance as the majority of the participants belong to the same subgroup at follow-up. However, this is only crucial when one wants to predict change of scores. This was not the central question of the current endeavor, as we were interested whether subgroup differences remained the same at the later measurement occasion. Fourthly, the modularity index indicated weakly defined communities. This has also been reported in other studies using community detection with similar types of data (Karalunas et al., 2014; Blanken et al., 2020). Since community detection and the modularity index are relatively new to psychological research, more methodological research is required into its properties with this type of data.

To our knowledge, the current study was the first to identify community detection-based subgroups in aging by inclusion of modifiable vulnerability and protective factors of aging. The study shows that people differ greatly in modifiable aging factors. Those with a low sense of mastery, high levels of social support, and high number of negative life events also had the lowest wellbeing and memory profile, currently and after 3 years. However, only a minority of participants belonged to this subgroup. Furthermore, transitions between subgroups are common. Therefore, healthy mental aging may be within reach of many, and even for those at risk, there seems to be considerable potential for improvement.

## Data Availability Statement

The data analyzed in this study is subject to the following licenses/restrictions: Dataset is available for research and can be requested by submitting a research proposal to the LASA Steering Group (see www.lasa-vu.nl for more info). The names of the datafiles used in the current study are described in the Supplementary Material (S1). Requests to access these datasets should be directed to www.lasa-vu.nl.

## Ethics Statement

The studies involving human participants were reviewed and approved by the Medical Ethics Committee of the Amsterdam University Medical Centers, Location VUmc (IRB numbers: 92/138, 2002/141, 2012/361, and 2016.301). The patients/participants provided their written informed consent to participate in this study.

## Author Contributions

TAR and JAAR: statistical analysis plan, first draft of the manuscript, feedback on subsequent versions of manuscript, and final approval of the manuscript. AALK and MH: study design LASA, feedback on analysis plan, feedback on subsequent versions of manuscript, and final approval of the manuscript. HMG: funding current study, statistical analysis plan, first draft of the manuscript, feedback on subsequent versions of manuscript, and final approval of the manuscript.

## Conflict of Interest

The authors declare that the research was conducted in the absence of any commercial or financial relationships that could be construed as a potential conflict of interest.

## Publisher’s Note

All claims expressed in this article are solely those of the authors and do not necessarily represent those of their affiliated organizations, or those of the publisher, the editors and the reviewers. Any product that may be evaluated in this article, or claim that may be made by its manufacturer, is not guaranteed or endorsed by the publisher.

## Footnotes

1 Although not preregistered, we exploratively calculated two standardized measures of subgroup similarity (see Supplementary Section 5).
