# Supplementary material for: Subgroups in Late Adulthood Are Associated With Cognition and Wellbeing Later in Life
Source: Front Psychol. 2021 Dec 1;12:780575. doi: 10.3389/fpsyg.2021.780575 (PMC8671814; doi:10.3389/fpsyg.2021.780575)
Supplement: Supplementary file 1 [file Data_Sheet_1.docx]

Supplementary materials for:

**Subgroups in Late Adulthood are Associated with Cognition and Wellbeing Later in Life**

Tulsi A. Radhoe^1^, Joost A. Agelink van Rentergem ^1^, Almar A. L. Kok^2,3^, Martijn Huisman^2,4^, Hilde M. Geurts^1,5^

^1^ Dutch Autism & ADHD Research Center (d’Arc), Department of Psychology, Brain & Cognition, University of Amsterdam, Nieuwe Achtergracht 129-B, 1018 WS Amsterdam, The Netherlands

^2^ Department of Epidemiology & Data Science, Amsterdam Public Health research institute, Amsterdam UMC – location VU University medical center, Amsterdam, the Netherlands

^3^ Amsterdam UMC, Vrije Universiteit Amsterdam, Department of Psychiatry, Amsterdam Public Health, Amsterdam, The Netherlands

^4^ Amsterdam UMC, Vrije Universiteit Amsterdam, Department of Sociology, Amsterdam Public Health, Amsterdam, The Netherlands

^5^ Dr. Leo Kannerhuis, Derkinderenstraat 10, 1062 DB Amsterdam, The Netherlands

**Table of content**

S1) Data files obtained from the Longitudinal Aging Study Amsterdam
S2) Measurement instruments for cluster variables and external validators

S3) Measurement instruments and corresponding descriptive analyses of subgroups identified by community detection analysis

S4) Attrition analyses

S5) Standardized measures of subgroup similarity

S6) Changes in cluster variable scores from T1 to T2
S7) References

**S1) Data files obtained from the Longitudinal Aging Study Amsterdam**

From measurement cycles G and H, the following data files were obtained from LASA for the current study: LASA021, LASA335, LASA135, LASA110, LASA036, LASA113, LASA313, LASA602, LASA702, LASA035, LASA235, LASA133, LASA333, LASA533, LASA025, LASA225, LASA026, LASA226, Z008, LASA027, LASA046, LASA272, LASA047, LASA247, LASA153, Z010, LASA011, LASA016, LASAd10, LASAd11, LASAd12, LASAd13, LASA152, LASA352, LASA179, LASA379, LASA156, LASA356.

From measurement cycle 2B, the following data files were requested: Z004, LASA022, LASA222.

**S2) Measurement instruments for cluster variables and external validators**

***Negative life* *events***

We evaluated negative life events with a selection of questions derived from the life event inventory (Tennant & Andrews, 1976). Participants reported whether they had experienced the following events in the past three years: a) death of a parent, sibling, child, or grandchild, b) illness of partner or other relative, c) being a victim of crime, d) having severe conflict, or e) having financial problems. This resulted in 12 variables, corresponding to each negative life event. A score of 1 indicated that the event had not occurred in the three years prior to the interview, and a score of 2 indicated that the event had occurred. These scores were recoded, such that 0 meant that the event had not occurred and 1 meant that the event had occurred. A sum score for these variables was calculated that ranged between 0 and 12. A lower score implied that the respondent experienced few of these negative life events and a higher score implied that he/she experienced more negative life events. Negative life events have strong associations with depressive symptoms and lower wellbeing (Kraaij et al., 2002). Although the number of experienced negative life events is not directly modifiable, its effect can be modified through interventions. In addition, modifiable resources such as social network, education and health status are negatively associated with the experience of negative life events later in life (Jopp & Schmitt, 2010).

***Alcohol use***

We asked respondents about the amount of days per week on which they drink alcohol and the number of alcoholic consumptions they drink each time. Responses on these two questions were multiplied, which resulted in an indication of the number of alcoholic consumptions per week. This value ranged between 0 and 77 (or more). A higher value indicates a higher number of alcoholic drinks per week (see for a similar approach Comijs, Aartsen, Visser, & Deeg, 2012; Pluijm et al., 2006). Alcohol use is related to memory problems and can be targeted in interventions (Heffernan, 2008; Mintzer, 2007; Platt et al., 2016).

***Physical activity***

Physical activity was assessed during an interview using the LASA Physical Activity Questionnaire (LAPAQ) (Stel et al., 2004). Participants reported how often and for how long they performed certain physical activities during the two weeks prior to the interview. These activities included walking outdoors, biking, gardening, light household activities and the two sport activities that were most frequently performed by the respondent. We calculated the total time (in minutes) during which a respondent was physically active during the past two weeks. In LASA, MET scores are often used to obtain an intensity-weighted total physical activity score (Caspersen et al., 1991). MET scores are calculated by including the type of activity, activity duration, but also a person’s body weight and biological sex. In the current study, we aim to reduce the influence of external variables on the variables we include for clustering. Therefore, we decided to include total physical activity in minutes rather than MET scores. Physical activity is associated with less cognitive decline and predicts wellbeing in older adults (Beydoun et al., 2014; Kadariya et al., 2019; McAuley et al., 2000) . Also, physical activity levels can be increased through interventions (Greaves et al., 2011; Müller-Riemenschneider et al., 2008).

***Sense of mastery***

Mastery refers to the extent to which respondents view themselves as being in control of the forces that affect their lifes in important ways (Pearlin et al., 1981). Mastery was assessed by the Pearlin Mastery Scale, which consists of seven items that were rated on a five-point scale that ranged from ‘strongly disagree’ to ‘strongly agree’ (Pearlin & Schooler, 1978). The instrument contains five questions that are negatively phrased and two positively phrased items. The negatively phrased items were reverse coded. We created a sum score that varied between 7 and 35, such that higher ratings corresponded to more feelings of mastery. The instrument’s reliability has shown to be reasonable to high with Cronbach’s α between 0.67 and 0.80 (Penninx et al., 1997; Peterson, 1999). In this study, the reliability was acceptable (α = 0.72). A high level of mastery, or stronger internal locus of control, is related to a better memory performance and higher wellbeing (Amrhein et al., 1999; Robinson & Lachman, 2017; Verhaeghen et al., 2000).

***Emotional and instrumental support received***

A domain-specific network delineation is employed that encompasses a detailed classification of personal relationships: household members, children and their partners, other family members, neighbors, contacts through work and school, members of associations, and other nonkin relationships (Van Tilburg, 1998). For each of these domains, respondents were asked to ‘Name the people you have frequent contact with and who are also important to you’.

For the nine relationships with the highest contact frequency, four questions about support exchanges were asked. The question for emotional support given was ‘How often in the last year did…tell you about his/her personal experiences and feelings?’ The question on received emotional support was ‘…did you tell…about your personal experiences and feelings?’ For instrumental support, the question was about help with daily chores in and around the house, such as preparing meals, cleaning the house, transportation, small repairs, and filling out forms. The answer categories were ‘never,’ ‘seldom,’ ‘sometimes,’ or ‘often’. Sum scores for emotional support received and instrumental support received were calculated that varied between 0 (low level of support) and 36 (high level of support). Leading a socially active life and receiving sufficient social support are related to a higher wellbeing later in life (Gerstorf et al., 2016; Yaffe et al., 2009). Interventions for social support can be effective in increasing one’s perceived level of social support (Hogan et al., 2002).

***Subjective wellbeing***

Subjective wellbeing is measured with three different questionnaires. First of all, satisfaction with life was measured by two questions defined by Van Zonneveld (1961). The first question asks participants about satisfaction with current life, and the second one about satisfaction with life as a whole. Both questions have five response categories that range from ‘very dissatisfied’ to ‘very satisfied’. A sum score was calculated that ranged from 2 (i.e. low satisfaction with life) to 10 (i.e. high satisfaction).

Second, the EuroQoL (EQ-5D) measures health-related quality of life (Brooks, 1996). The questionnaire consists of five questions and a visual analog scale. Each question represents one the following domains: mobility, self-care, usual activities, pain/discomfort, and anxiety and depression. Response categories vary according to the specific question, but they can roughly be characterized as having ‘no problems’, ‘some problems’, and ‘extreme problems’. The responses on these items were converted into a weighted health state index according to the Time Trade OFF method (Dolan, 1997). An index score of 0 indicates death and a score of 1 indicates perfect health. The internal consistency of the EQ-5D is acceptable with Cronbach’s *α* ranging between 0.63 and 0.73 (De Smedt et al., 2013; Khanna et al., 2013), and the test-retest reliability is moderate (intraclass correlation coefficient = 0.6) (Sonntag et al., 2013). In the current study, the internal consistency was acceptable (*α* = 0.78).

Third, we measured functional health and wellbeing by the Short Form 12 (SF-12) health survey, which is a subset of the larger SF-36 (Ware et al., 1996). This instrument was used to measure the following eight health aspects: physical functioning, role limitations due to physical health problems, bodily pain, general health, vitality, social functioning, role limitations due to emotional problems, and mental health. Sum scores were calculated for two summary scales of the SF-12, the Physical Component Summary (PCS) and the Mental Component Summary (MCS). These summary scales have a mean value of 50 and a standard deviation of 10. The test-retest reliability of the instrument is high, with *r* = 0.760 to 0.864, and the internal consistencies of the MSC and PCS are high with Cronbach’s *α* higher than 0.80 (Hayes et al., 2017). In the current study, the internal consistency of MSC and PCS was questionable (*α* = 0.55).

**S3) Measurement instruments and corresponding descriptive analyses of subgroups identified by community detection analysis**

***Age***: In order to better characterize the identified subgroups, we checked whether there was a significant mean age difference between the subgroups by performing an ANOVA with subgroup membership as the independent variable and age as the dependent variable.

***Gender****:* To investigate whether the distribution of males and females differed across subgroups, we used a Pearson’s chi-square test with gender (two categories) and subgroup membership as categorical variables.

***Country of origin****:* Differences in country of origin across subgroups were also investigated by means of a Pearson’s chi-square test with country of origin (Netherlands versus ‘other’) and subgroup membership as categorical variables.

***Medication use:*** Differences between subgroups in the number of individuals who use medication was investigated by a Pearson’s chi-square test in which medication use (i.e. yes or no) and subgroup membership were entered as variables. Furthermore, differences in medication use were analyzed by an ANOVA in which the number of medicines used was the dependent variable and subgroup membership the independent variable.

***Marital status****:* We checked whether subgroups differed in marital status by a Pearson’s chi-square test with marital status (i.e. unmarried, married, divorced, widowed) and subgroup membership as categorical variables.

***Household composition****:* To assess whether household composition (i.e. number of other persons in household) differed across subgroups, we performed an ANOVA with subgroup membership as independent variable.

***Depression diagnosis:*** The Composite International Diagnostic Interview (CIDI) (Robins et al., 1988) was used to assess diagnoses of mental disorders based on Diagnostic and Statistical Manual of Mental Disorders-IV (American Psychiatric Association, 2000). Subgroup differences in depression diagnosis were investigated by a Pearson’s chi-square test. Differences in depression or anxiety diagnoses were investigated by two Pearson’s chi-square tests. It should be noted that the CIDI was only administered to a subsample of participants (N=266), which caused a relatively high number of missing values in these analyses.

***Anxiety diagnosis****:* Anxiety diagnosis was also assessed by the CIDI (Robins et al., 1988). Subgroup differences in anxiety diagnoses were investigated by a Pearson’s chi-square test.

***ADHD-score****:* ADHD symptoms were assessed by the ADHD screening list (Barkley et al., 2007). This questionnaire consists of seven items with two response categories (i.e., ‘yes’ or ‘no’). We used an ANOVA to investigate differences in total scores on the ADHD screening list between subgroups.

***Fluid intelligence:*** The Raven Coloured Progressive Matrices (RCPM) were used to assess fluid intelligence (Raven, 1995). The RCPM consists of three sections, namely A, Ab and B. Each subset consists of 12 items. The items consist of a matrix from which one section is missing. The respondent has to select the missing section among six alternatives that are printed below the matrix. A correct response is scored as one point, which results in a score ranging between 0 and 12 per subset. In LASA, only subset A and B were administered. Differences in fluid intelligence scores across subgroups were analyzed by an ANOVA with Raven sum scores for subset A and B separately, as dependent variables and subgroup membership as independent variable.

**S4) Attrition analyses**
The attrition from T1 to T2 is depicted in sFigure 1. We performed six ANOVAs and six Pearson’s chi-square tests on descriptive variables to compare differences between the group not analyzed at T2 (N=326) and the group analyzed at both T1 and T2 (N=1152). Results are presented in sTable 1.

The group of participants who dropped out and were, therefore, not analyzed at T2 (i.e., attrition group), was older than the group that was measured at T1 and T2 (i.e., included group). An explanation for this difference is that older age is more likely to be associated with drop-out due to fluctuating health status or death. The attrition group also had fewer household members, took a higher number of medicines and had lower scores both Raven subtests as compared to the included group. Also, this attrition group reported more medication use, and had fewer married participants that the group who participated at both T1 and T2. Furthermore, the attrition group, contained more participants from Subgroup C (45%), and fewer participants from Subgroup A and Subgroup B (i.e., 26% and 29%, respectively).


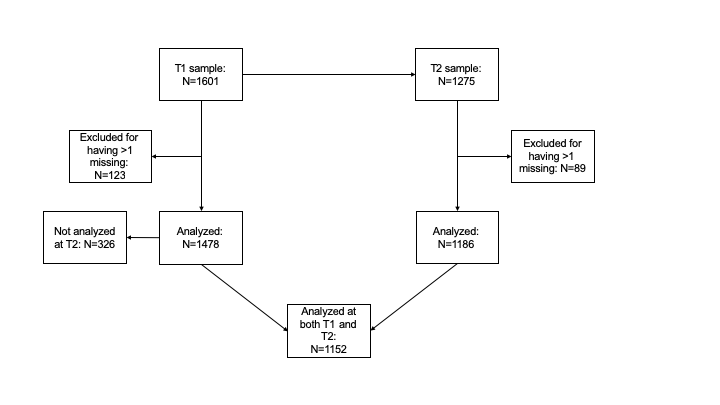


*sFigure 1.* Attrition from T1 to T2

sTable 1
*Raw scores on descriptive variables at T1 for the group that was not analyzed at T2 (attrition group; N=326) and the group that was analyzed at both T1 and T2 (included group; N=1152).*

|  | **Group** | |  |
| --- | --- | --- | --- |
|  | Not analyzed at T2  (attrition) | Analyzed at T1 and T2  (included) |  |
| Variable | N = 326 | N = 1152 | test statistic(*df*) |
| **Descriptive variables** |  |  |  |
| Age *M*(*SD*) | 77.71 (9.06) | 71.50 (7.52) | *F*(1,1476)= 157.60*** |
| # household members *M*(*SD*) | 0.61 (0.57) | 0.77 (0.60) | *F*(1,1447)= 17.30*** |
| # medicines *M*(*SD*) | 4.43 (3.20) | 2.98 (2.76) | *F*(1,1415)= 59.89*** |
| Raven A-score *M*(*SD*) | 9.17 (3.02) | 10.40 (1.94) | *F*(1,1415)= 72.36*** |
| Raven B-score *M*(*SD*) | 6.57 (3.40) | 8.72 (2.73) | *F*(2,1415)= 129.10*** |
| ADHD-score *M*(*SD*) | 0.64 (1.17) | 0.58 (1.21) | *F*<1 |
| Gender |  |  | χ^2^(1) = 0.03 |
| N_male_ (%) | 147 (45) | 528 (46) |  |
| N_female_(%) | 179 (55) | 624 (54) |  |
| Country of Origin |  |  | χ^2^(1) = 0.40 |
| N_Netherlands_ (%) | 323 (99) | 1147 (100) |  |
| N_Other_ (%) | 3 (1) | 5 (0) |  |
| Current depression |  |  | χ^2^(1) = 0.72 |
| N_Yes_ (%) | 2 (25) | 20 (9) |  |
| N_No_ (%) | 6 (75) | 193 (91) |  |
| Lifetime depression |  |  | χ^2^(1) < 0.01 |
| N_Yes_ (%) | 2 (25) | 49 (23) |  |
| N_No_ (%) | 6 (75) | 164 (77) |  |
| Lifetime anxiety |  |  | χ^2^(1) = 0.24 |
| N_Yes_ (%) | 1 (14) | 57 (30) |  |
| N_No_ (%) | 6 (86) | 131 (70) |  |
| Medication use |  |  | χ^2^(1) = 14.83*** |
| N_Yes_ (%) | 257 (89) | 883 (78) |  |
| N_No_ (%) | 33 (11) | 244 (22) |  |
| Marital status |  |  | χ^2^(3) = 35.80*** |
| N_never married_ (%) | 11 (3) | 62 (5) |  |
| N_married_ (%) | 168 (52) | 761 (66) |  |
| N_divorced_ (%) | 29 (9) | 89 (8) |  |
| N_widowhood_ (%) | 118 (36) | 240 (21) |  |
| Subgroup membership at T1 |  |  | χ^2^(2) = 9.83** |
| N_Subgroup A_ (%) | 83 (26) | 352 (30) |  |
| N_Subgroup B_ (%) | 96 (29) | 390 (34) |  |
| N_Subgroup C_ (%) | 147 (45) | 410 (36) |  |

*Note.* ** p* < 0.05*, ** p* < 0.01*, *** p* < 0.001

**S5) Standardized measures of subgroup similarity**

Although not preregistered, we exploratively calculated two measures of subgroup similarity between the subgroups identified at T1 and T2. First, we calculated the Rand Index (RI) that ranges between 0 and 1 (Rand, 1971). Second, we calculated the Hubert-Arabie Adjusted Rand Index (ARI_HA_; Hubert & Arabie, 1985), which ranges between -1 and 1. For both measures, values greater than 0.90 can be interpreted as excellent subgroup recovery (i.e. similarity between subgroups at T1 and T2) and values less than 0.65 reflect poor recovery (Steinley, 2004).

In the current study, results indicated a poor recovery of subgroups from T1 to T2 (RI = 0.60, ARI_HA_ = 0.10). However, it should be noted that a perfect similarity between subgroups at two measurement occasions is unlikely, since individuals are subject to change. A perfect recovery would imply that there are no transitions between subgroups (i.e., people do not change at all over time), which is not likely or clinically desirable. These measures are less applicable to the current study, and therefore, one should consider the underlying profiles of subgroups across measurement occasions as well.

**S6) Changes in cluster variable scores from T1 to T2**

**
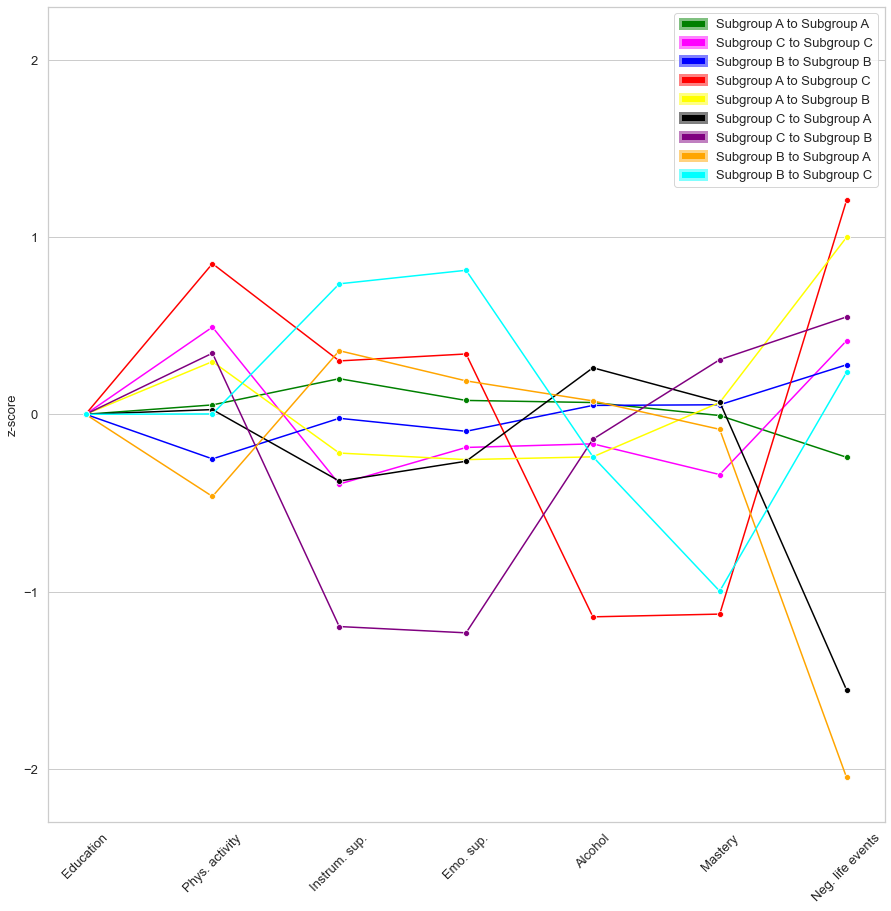
**

*sFigure 2.* Changes in mean z-scores on cluster variables from T1 to T2.

*Note*. Scores as shown as z-scores based on the total sample mean. A z-score above 0 indicates an increase from T1 to T2, whereas a z-score below 0 indicates a decrease. A z-score of 0 indicates absence of change.

**S7) References**

American Psychiatric Association. (2000). *Diagnostic and statistical manual of mental disorders* (4th ed.). Author.

Amrhein, P. C., Bond, J. K., & Hamilton, D. A. (1999). Locus of control and the age difference in free recall from episodic memory. *Journal of General Psychology*, *126*(2), 149–164. https://doi.org/10.1080/00221309909595358

Barkley, R. A., Murphy, K. R., & Fischer, M. (2007). *ADHD in adults: What the science says*. Guilford.

Beydoun, M. A., Beydoun, H. A., Gamaldo, A. A., Teel, A., Zonderman, A. B., & Wang, Y. (2014). Epidemiologic studies of modifiable factors associated with cognition and dementia: Systematic review and meta-analysis. *BMC Public Health*, *14*(1), 1–33. https://doi.org/10.1186/1471-2458-14-643

Brooks, R. (1996). EuroQol: the current state of play. *Health Policy*, *37*(1), 53–72. https://doi.org/10.1016/0168-8510(96)00822-6

Caspersen, C. J., Bloemberg, B. P. M., Saris, W. H. M., Merritt, R. K., & Kromhout, D. (1991). The prevalence of selected physical activities and their relation with coronary heart disease risk factors in elderly men: The Zutphen Study, 1985. *American Journal of Epidemiology*, *133*(11), 1078–1092.

Comijs, H. C., Aartsen, M. J., Visser, M., & Deeg, D. J. H. (2012). Alcoholgebruik onder 55-plussers in Nederland. *Tijdschrift Voor Gerontologie En Geriatrie*, *43*(3), 115–126. https://doi.org/10.1007/s12439-012-0018-9

De Smedt, D., Clays, E., Doyle, F., Kotseva, K., Prugger, C., Pająk, A., Jennings, C., Wood, D., & De Bacquer, D. (2013). Validity and reliability of three commonly used quality of life measures in a large European population of coronary heart disease patients. *International Journal of Cardiology*, *167*(5), 2294–2299. https://doi.org/10.1016/j.ijcard.2012.06.025

Dolan, P. (1997). Modeling valuations for EuroQol health states. *Medical Care*, *35*(11), 1095–1108. https://doi.org/10.1097/00005650-199711000-00002

Gerstorf, D., Hoppmann, C. A., Löckenhoff, C. E., Infurna, F. J., Wagner, G. G., & Ram, N. (2016). Terminal Decline in Well-Being: The Role of Social Orientation. *Psychology and Aging*, *31*(2), 149–165. http://dx.doi.org/10.1037/pag0000072

Greaves, C. J., Sheppard, K. E., Abraham, C., Hardeman, W., Roden, M., Evans, P. H., & Schwarz, P. (2011). Systematic review of reviews of intervention components associated with increased effectiveness in dietary and physical activity interventions. *BMC Public Health*, *11*. https://doi.org/10.1186/1471-2458-11-119

Hayes, C. J., Bhandari, N. R., Kathe, N., & Payakachat, N. (2017). Reliability and validity of the medical outcomes study short form-12 version 2 (SF-12v2) in adults with non-cancer pain. *Healthcare*, *5*(2), 1–12. https://doi.org/10.3390/healthcare5020022

Heffernan, T. M. (2008). The impact of excessive alcohol use on prospective memory: A brief review. *Current Drug Abuse Reviews*, *1*(1), 36–41. https://doi.org/10.2174/1874473710801010036

Hogan, B., Linden, W., & Najarian, B. (2002). Social support interventions: Do they work? *Clinical Psychology Review*, *22*, 381–440. https://doi.org/10.1016/S0272-7358(01)00102-7

Hubert, L., & Arabie, P. (1985). Comparing partitions. *Journal of Classification*, *2*, 193–218. https://doi.org/10.1007/BF01908075

Jopp, D. S., & Schmitt, M. (2010). Dealing with negative life events: Differential effects of personal resources, coping strategies, and control beliefs. *European Journal of Ageing*, *7*(3), 167–180. https://doi.org/10.1007/s10433-010-0160-6

Kadariya, S., Gautam, R., & Aro, A. R. (2019). Physical Activity, Mental Health, and Wellbeing among Older Adults in South and Southeast Asia: A Scoping Review. *BioMed Research International*, *2019*. https://doi.org/10.1155/2019/6752182

Khanna, R., Jariwala, K., & Bentley, J. P. (2013). Psychometric properties of the EuroQol Five Dimensional Questionnaire (EQ-5D-3L) in caregivers of autistic children. *Quality of Life Research*, *22*(10), 2909–2920. https://doi.org/10.1007/s11136-013-0423-8

Kraaij, V., Arensman, E., & Spinhoven, P. (2002). Negative life events and depression in elderly persons: A meta-analysis. *Journals of Gerontology - Series B Psychological Sciences and Social Sciences*, *57*(1), 87–94. https://doi.org/10.1093/geronb/57.1.P87

McAuley, E., Blissmer, B., Marquez, D. X., Jerome, G. J., Kramer, A. F., & Katula, J. (2000). Social relations, physical activity, and well-being in older adults. *Preventive Medicine*, *31*(5), 608–617. https://doi.org/10.1006/pmed.2000.0740

Mintzer, M. Z. (2007). The acute effects of alcohol on memory: A review of laboratory studies in healthy adults. *International Journal on Disability and Human Development*, *6*(4), 397–403. https://doi.org/10.1515/IJDHD.2007.6.4.397

Müller-Riemenschneider, F., Reinhold, T., Nocon, M., & Willich, S. N. (2008). Long-term effectiveness of interventions promoting physical activity: A systematic review. *Preventive Medicine*, *47*(4), 354–368. https://doi.org/10.1016/j.ypmed.2008.07.006

Pearlin, L. I., Menaghan, E. G., Lieberman, M. A., Joseph, T., Lieberman, M. A., & Mullan, J. T. (1981). The stress process. *Journal of Health and Social Behavior*, *22*(4), 337–356. https://doi.org/10.2307/2136676

Pearlin, L. I., & Schooler, C. (1978). The structure of coping. *Journal of Health and Social Behavior*, *19*(1), 2–21. https://doi.org/10.2307/2136319

Penninx, B. W. J. H., Kriegsman, D. M. W., Boeke, A. J. P., Van Eijk, J. T. M., Van Tilburg, T., & Deeg, D. J. H. (1997). Effects of social support and personal coping resources on mortality in older age: The longitudinal aging study Amsterdam. *American Journal of Epidemiology*, *146*(6), 510–519. https://doi.org/10.1093/oxfordjournals.aje.a009305

Peterson, C. L. (1999). *Stress at work. A sociological perspective*. CRC Press.

Platt, L., Melendez-Torres, G. J., O’Donnell, A., Bradley, J., Newbury-Birch, D., Kaner, E., & Ashton, C. (2016). How effective are brief interventions in reducing alcohol consumption: do the setting, practitioner group and content matter? Findings from a systematic review and metaregression analysis. *BMJ Open*, *6*(8), e011473. https://doi.org/10.1136/bmjopen-2016-011473

Pluijm, S. M. F., Smit, J. H., Tromp, E. A. M., Stel, V. S., Deeg, D. J. H., Bouter, L. M., & Lips, P. (2006). A risk profile for identifying community-dwelling elderly with a high risk of recurrent falling: results of a 3-year prospective study. *Osteoporosis International*, *17*(3), 417–425. https://doi.org/10.1007/s00198-005-0002-0

Rand, W. M. (1971). Objective criteria for the evaluation of clustering methods. *Journal of the American Statistical Association*, *66*(336), 846–850. https://doi.org/10.1080/01621459.1971.10482356

Raven, J. C. (1995). *Manual for the Coloured Progressive Matrices (revised)*. NFRE-Nelson.

Robins, L. N., Wing, J., Wittchen, H. U., Helzer, J. E., Babor, T. F., Burke, J., Farmer, A., Jablenski, A., Pickens, R., & Regier, D. A. (1988). The Composite International Diagnostic Interview. An epidemiologic Instrument suitable for use in conjunction with different diagnostic systems and in different cultures. *Archives of General Psychiatry*, *45*(12), 1069–1077. https://doi.org/10.1001/archpsyc.1988.01800360017003

Robinson, S. A., & Lachman, M. E. (2017). Perceived control and aging: A mini-review and directions for future research. *Gerontology*, *63*(5), 435–442. https://doi.org/10.1159/000468540

Sonntag, M., Konnopka, A., Leichsenring, F., Salzer, S., Beutel, M. E., Herpertz, S., Hiller, W., Hoyer, J., Joraschky, P., Nolting, B., Pöhlmann, K., Stangier, U., Strauss, B., Willutzki, U., Wiltink, J., Leibing, E., & König, H. H. (2013). Reliability, validity and responsiveness of the EQ-5D in assessing and valuing health status in patients with social phobia. *Health and Quality of Life Outcomes*, *11*, 1–9. https://doi.org/10.1186/1477-7525-11-215

Steinley, D. (2004). Properties of the Hubert-Arabie adjusted rand index. *Psychological Methods*, *9*(3), 386–396. https://doi.org/10.1037/1082-989X.9.3.386

Stel, V. S., Smit, J. H., Pluijm, S. M. ., Visser, M., Deeg, D. J. H., & Lips, P. (2004). Comparison of the LASA physical activity questionnaire with a 7-day diary and pedometer. *Journal of Clinical Epidemiology*, *57*(3), 252–258. https://doi.org/10.1016/j.jclinepi.2003.07.008

Tennant, C., & Andrews, G. (1976). A scale to measure the stress of life events. *Australian & New Zealand Journal of Psychiatry*, *10*(1), 27–32. https://doi.org/10.3109/00048677609159482

Van Tilburg, T. (1998). Losing and gaining in old age: Changes in personal network size and social support in a four-year longitudinal study. *Journals of Gerontology - Series B Psychological Sciences and Social Sciences*, *53*(6), 313–323. https://doi.org/10.1093/geronb/53B.6.S313

Van Zonneveld, R. J. (1961). *The health of the aged*. Van Gorcum.

Verhaeghen, P., Geraerts, N., & Marcoen, A. (2000). Memory complaints, coping, and well-being in old age: A systemic approach. *Gerontologist*, *40*(5), 540–548. https://doi.org/10.1093/geront/40.5.540

Ware, J. E., Kosinski, M., & Keller, S. D. (1996). A 12-Item short-form health survey: Construction of scales and preliminary tests of reliability and validity. *Medical Care*, *34*(3), 220–233. https://doi.org/10.1097/00005650-199603000-00003

Yaffe, K., Fiocco, A. J., Lindquist, K., Vittinghoff, E., Simonsick, E. M., Newman, A. B., Satterfield, S., Rosano, C., Rubin, S. M., Ayonayon, H. N., & Harris, T. B. (2009). Predictors of maintaining cognitive function in older adults: The Health ABC Study. *Neurology*, *72*(23), 2029–2035. https://doi.org/10.1212/WNL.0b013e3181a92c36
